# Editorial: Physical culture from an interdisciplinary perspective

**DOI:** 10.3389/fpsyg.2023.1254027

**Published:** 2023-07-25

**Authors:** Paweł Adam Piepiora, Zbigniew Norbert Piepiora, Daniela Stackeová, Justyna Bagińska

**Affiliations:** ^1^Faculty of Physical Education and Sports, Wroclaw University of Health and Sport Sciences, Wrocław, Poland; ^2^Faculty of Environmental Engineering and Geodesy, Wrocław University of Environmental and Life Sciences, Wrocław, Poland; ^3^Department of Pedagogy and Psychology, College of Physical Education and Sport Palestra, Prague, Czechia; ^4^Wrocław Business University of Applied Sciences, Wrocław, Poland

**Keywords:** physical education, physiotherapy, sport, tourism, recreation

## About physical culture sciences

Physical culture is the totality of activities according to the rules and norms of behavior accepted in a given social environment. These activities are aimed at health-promoting values. The result is correct psycho-physical development of an individual through the outcomes of these activities. One can participate in physical culture through five areas: physical education, physiotherapy, sport, tourism, and recreation. The area of physical education should be understood as guiding people's physical development and health through physical activity, developing fitness and efficiency and shaping their correct posture, as well as prosocial attitudes as part of school education. The area of physiotherapy, on the other hand, aims to restore fitness and function in everyday life for people affected by injury, illness or disability through exercise and physical therapy treatments. The area of sport, on the other hand, is concerned with all forms of physical activity which, through casual or organized participation of sporting persons, are aimed at achieving results in competition at all levels according to specific regulations. The next area, tourism, refers to all activities that aim to explore the world's resources and is carried out by traveling physically active way. The last area is recreation, which refers to all forms of physical activity undertaken for leisure, psycho-physical renewal and relaxation. As a result of the above, it has been concluded that the physical culture sciences are concerned with the study of the stimulation of the human organism, the adaptation of this organism to the environment, the compensation of its deficits and the correction of its postural defects. Therefore, it is possible to state conclusions in this discipline of sciences about the values of the body in the health, utilitarian, aesthetic, hedonistic and agonistic sense.

## Directions for integrating psychology into physical culture sciences

Psychology is interdisciplinary because it is important in every aspect of social life. Psychology also has a place in the field of physical culture. In the curricula of these studies, there are specialized subjects in the field of psychology: physical activity (concerning physical education), rehabilitation (concerning physiotherapy), sport (concerning sport), tourism (concerning tourism), and leisure time (concerning recreation). In this sense, psychology and physical culture have the same goal, which is health-promoting values that translate into proper psychophysical development of the society. The above justifies the advisability of editing a Research Topic of Frontiers in Psychology under the title “*Physical culture from an interdisciplinary perspective*”. For this reason, the editors aimed at presenting the results of research in physical culture sciences from various scientific disciplines the issues of which are in the curricula of studies in physical education, physiotherapy, sport, tourism and recreation. The interdisciplinary view of 21st century problems points to the important role of physical culture as an element of social life.

## Contents of the Research Topic

In the first paper, Prokopczyk and Wochyński verified the effect of the training process with the use of the Special Aviation Gymnastics Instruments (SAGI) on the improvement of psychomotor ability among cadets, expressed as an increase in the percentage ability to perform all tasks and the number of coils per loop. It was found that the training process on the SAGI raised the psychomotor level of the cadets. This study was a pilot study.

In the next paper, Ma analyzed the case of the Shanghai municipality to understand the complexity of the change in elite sports at the provincial level. Based on semi-structured face-to-face interviews and official and semi-official documents, information on strategies for reviewing sport policy directions was provided. Guangdong was found to serve as a powerful impetus for elite sports policy in Shanghai. It is reinforced by the activities of Shanghai University of Sports. And the general director of the Shanghai Sports Administration plays a key role in promoting sport policy proposals.

In the third paper, Zhigang et al. presented the factors influencing significant sport consumption and the mechanism of its impact. Based on their study in China, they found that social needs have a significant positive effect on sports consumption behavior through the mediating effect of team membership and the drive for self-esteem. However, self-enhancement motivation does not have a mediating effect on the relationship between social needs and sport consumption.

In the fourth paper, Montt-Blanchard et al. presented issues of designing opportunities to manage the barriers imposed by type 1 diabetes (T1D) that were gained during marathon running. Six insights related to T1D self-management were identified and analyzed in relation to the associated design tools. Reference was made to the important role of physical activity in crossing human boundaries and the need for further research in physical culture and health psychology.

In the following paper, Jaworski, Lech, Witkowski et al. determined the influence of training and selection on postural stability and its relationship with sport level in judo practitioners aged 11–14 years. Their balance was assessed using the CQ Stab 2P stabilographic platform (CQ Elektronik System, Poland). It was found that the mean frequency (MF) of center of pressure (COP) was significantly higher in judokas than in non-trainees. The correlations between the other values of the parameters characterizing balance level and sport level were statistically insignificant.

In the following article, Siekanska et al. examined the validity of the Polish Short Form version of the Self-Regulated Learning-Sport Practice survey among competitive athletes. They analyzed factor validity and reliability, criterion validity, and convergent validity of the Polish Short Form survey. Athletes at amateur, regional, national, and international elite levels completed the survey, along with concurrent subscales: General Self-Efficacy Scale (GSES); Metacognitive Self-Scale (MS-24); Action Control Scale (ACS-90). Based on strong criterion-relevance and moderate evidence of convergent validity, the Polish Short Form of the Self-Regulated Learning-Sport Practice survey was found to be a promising tool for use in Polish sport and is subject to further validation.

In the seventh paper, Piepiora et al. compared personalities of Polish mountaineers. For this purpose, they surveyed a population of male and female Polish mountaineers: Alpine climbers and Himalayan climbers. The Big Five model was used, including the NEO-FFI Personality Questionnaire. It was found that a significant difference between the personalities of Polish Alpine climbers and Polish Himalayan climbers in terms of agreeableness was found only among women: female Alpine climbers are more agreeable than female Himalayan climbers, which may imply ethical dilemmas in the high mountains.

In another paper, Mackala et al. determined the impact of marathon performance on muscle stiffness in runners over 50 years of age. Thirty-one long-distance runners aged 50–73 years participated in the experiment. Quadriceps and calf muscle stiffness were measured using a Myoton device in two independent tests: the day prior to the marathon and 30 min after the completion of a marathon run. Reduced muscle stiffness was found, but only in the triceps calf muscle in the dominant (left) leg. The research should be continued and an optimal evaluation should also address direct and indirect analyses of running economy, running technique, and HRmax and VO_2_max variables.

In the following paper, Bibrowicz et al. determined the asymmetry of the pelvis in Polish young adults aged 19–29. The prevalence of spatial asymmetry of the pelvis was analyzed based on the author's clinical classification and the significance of body weight and height for the asymmetries analyzed on a sample of 300 young adults. Asymmetries in the pelvis area were observed in less than three-quarters of the examined population. Oblique pelvis was found in less than a quarter of women and in more than one-third men with the predominant structural asymmetries. Rotated pelvis was observed in more than one third of women and men with dominating functional asymmetries.

In the 10th paper, Jaworski, Lech, Żak et al. studied the relationships between selected indices of postural stability and sports performance in elite badminton players. The pilot study examined 10 elite players from the Polish national badminton team. The scope of the study included basic somatic characteristics such as body height, body weight, BMI and training experience. The Microgate GYKO inertial sensor system was used to assess the postural stability of the athletes. The athletes with higher ranking positions were found to have higher levels of postural stability in the tests. Furthermore, higher correlation coefficient values were found for the test performed in single-leg standing. This indicates that special attention should be paid to the development of levels of postural stability in order to improve sports performance among badminton players.

In the last paper Stackeová et al. did a study of a modification of EAT-26 questionnaire to detect pathological eating behavior in competitive athletes. On the basis of the EAT-26 questionnaire, a modification was created for use among professional athletes. It was then validated among athletes in aesthetic sports with a sample of 100 respondents aged 16–26 years. Five strong factors were identified: diet control, weight control, training obsession, appetite, calorie counting; which can be defined as significant factors that influence the onset of disordered eating behavior or the subsequent development of eating disorders. Respondents from the bodybuilding and fitness sector scored highest on average. It was concluded that disordered eating behaviors and eating disorders are being hidden in the sporting environment and that diagnosis in this environment is difficult.

## Author contributions

PP: Conceptualization, Data curation, Formal analysis, Funding acquisition, Investigation, Methodology, Project administration, Resources, Software, Supervision, Validation, Visualization, Writing—original draft, Writing—review and editing. ZP: Conceptualization, Data curation, Formal analysis, Funding acquisition, Investigation, Methodology, Project administration, Resources, Software, Supervision, Validation, Visualization, Writing—original draft, Writing—review and editing. DS: Conceptualization, Data curation, Formal analysis, Funding acquisition, Investigation, Methodology, Project administration, Resources, Software, Supervision, Validation, Visualization, Writing—original draft, Writing—review and editing. JB: Conceptualization, Data curation, Formal analysis, Funding acquisition, Investigation, Methodology, Project administration, Resources, Software, Supervision, Validation, Visualization, Writing—original draft, Writing—review and editing.

